# Placental Transfer of Maternal Vaccine-Induced Antibodies Differentially Shapes Fc Receptor Engagement, Antibody Function, and Infant Immunity

**DOI:** 10.21203/rs.3.rs-10959044/v1

**Published:** 2026-10-06

**Authors:** Maclaine Parish, Rishi Pandey, Anna Yin, Sattya Talukdar, Han-Sol Park, Olyvia Jasset, Laura Ibanez Pintor, Rachel Yinger, Sophia Feinerman, Caroline Bald, Zhaojing Liu, Alison Araten, Lael Yonker, Andrew Pekosz, Andrea Edlow, Sabra Klein

**Affiliations:** Johns Hopkins University; Johns Hopkins University; Johns Hopkins University; Johns Hopkins University; Johns Hopkins University; Mass General Brigham; Mass General Brigham; Mass General Brigham; Mass General Brigham; Mass General Brigham; Mass General Brigham; Mass General Brigham; The University of Texas Southwestern Medical Center; Johns Hopkins University; Mass General Brigham; Johns Hopkins University

**Keywords:** influenza, respiratory syncytial virus, pregnancy, placental antibody transfer, Fc-mediated effector functions, systems serology

## Abstract

Maternal vaccination protects mothers directly and infants indirectly through placental transfer of vaccine-induced antibodies. Using multidimensional systems serology across matched maternal, cord blood, and two-month infant samples, we evaluated virus-specific antibody isotypes and functions following maternal influenza and RSV vaccination. The predominant subclass transferred and maintained in infants against H1N1 hemagglutinin (H1) and RSV prefusion (Pre)F was IgG1. Transferred RSV PreF- and H1-specific antibodies exhibited increased neutralization, Fcγ receptor-binding, antibody-dependent complement deposition, and cellular phagocytosis compared with non-transferred maternal antibodies. Although total antibody-dependent neutrophil phagocytosis (ADNP) was comparable between maternal and cord blood for RSV PreF antibodies, RSV PreF, but not H1-specific, FcγRIII-binding antibodies associated with neutrophil phagocytic activity were selectively transferred. Predictive modeling identified distinct correlates of antigen-specific antibody persistence, with maternal and cord blood estradiol emerging as a shared contributor to higher infant IgG1 through two months of age, suggesting estrogenic potentiation of antibody responses in early life.

## Introduction

Respiratory viral infections are a leading cause of severe illness and death among children under five years of age. Globally, respiratory syncytial virus (RSV) and influenza viruses contribute to approximately 3.3 million^1–3^ and 870,000 ^4^ child hospitalizations, respectively, each year. The severity of viral respiratory infections during early infancy is driven by the immaturity of the neonatal immune system which predisposes infants to airway obstruction from inflammation, edema, and mucus accumulation^5–7^. These effects are exacerbated in infants with comorbidities, including low birth weight and prematurity ^8–12^. Although vaccines are available for some respiratory pathogens, infants are typically not eligible for vaccination against respiratory pathogens until at least six months of age^13,14^, leaving them vulnerable during a period of heightened susceptibility. To bridge this gap, passive immunization strategies have been developed to provide protection during early life^15^. While monoclonal antibodies provide protection through administration of a single neutralizing IgG1 antibody^16–18^, maternal vaccination transfers a diverse polyclonal repertoire of vaccine-induced antibodies to the fetus, providing an opportunity to confer both neutralizing and non-neutralizing humoral immunity during early life.

Maternal immunization is a public health strategy for protecting young infants against pathogens for which direct vaccination is ineffective, unavailable, or delayed until later in infancy ^13^. Maternal antibodies, predominantly IgG, are transplacentally transferred to the fetus through an active and highly regulated process, mediated by the neonatal FcR (FcRn) as well as other non-canonical Fc receptors, rather than by passive diffusion^19^. Extent of transfer varies substantially among IgG subclasses, with IgG1 transferred most efficiently, followed by IgG3, IgG4, and IgG2^20^. While mammary tissue expresses FcRn and some IgG is transferred through breastfeeding, breastmilk is 90% IgA antibodies, followed by small contributions of IgM and IgG^21^.

For many viral vaccines, a correlate of protection has not been conclusively identified, but is often presumed to reflect the transfer of neutralizing antibodies^22–24^. However, there is growing appreciation that neutralization alone does not fully explain protection, particularly for pathogens such as RSV that lack a defined correlate of protection^25–28^. Increasing evidence indicates that Fc-mediated effector functions, including antibody-dependent cellular phagocytosis (ADCP), antibody-dependent neutrophil phagocytosis (ADNP), antibody-dependent complement deposition (ADCD), and antibody-dependent NK cell cytotoxicity (ADNK), contribute to antiviral immunity through the clearance of infected cells and opsonized virions^29–33^.

Systems serology has emerged as a powerful tool to characterize the breadth and quality of vaccine-induced antibody responses by integrating measurements of virus neutralization with antibody isotypes, Fc receptor (FcR) binding, and Fc-mediated effector functions^34,35^. Inflammatory cytokines, steroid hormones, and other maternal-fetal immune factors may influence antibody glycosylation, FcR interactions, and innate immune cell activity, thereby shaping placental antibody transfer^36–39^. Applying systems serology approaches within the context of maternal vaccination may provide insight into how both antibody-intrinsic features and the maternal-fetal immune environment shape the functional antibody repertoire delivered to infants and influence the durability of passive immunity in early life.

Studying a cohort of pregnant women who received influenza and RSV vaccines in 2022 during the first season of RSV vaccine availability in pregnancy, we determined how maternal vaccination against RSV, administered any time from 32–36 weeks’ gestation^40,41^, and influenza, administered any time during pregnancy, shaped the antibody repertoire in maternal blood, cord blood, and 2 month infant samples. By integrating measurements of antibody isotypes, Fc receptor binding, neutralization, Fc-mediated effector functions, cytokines, and steroid hormones, we sought to define the features of vaccine-induced antibodies that are preferentially transferred across the placenta and associated with sustained neonatal immunity.

## Results

### Patient demographics and vaccinations

A cohort of pregnant women (n = 31; Table 1) receiving the Abrysvo RSV vaccine during the third trimester and the seasonal quadrivalent (QIV) influenza vaccine during any trimester, and their infants, were enrolled from October 2023 to May 2024 (MGB IRB protocols # 2023P001874 and #2020P000955, see Methods for additional details). The cohort reflected a mean maternal age of 35.6 ± 4.37 years, full-term pregnancies with appropriate fetal growth. 45% of the infants (14/31) were female. Study demographics are described in Table 1. All 31 participants received RSV, influenza and Tdap vaccines in pregnancy, and 20/31 also received COVID-19 boosters. Mean gestational age at vaccination was greater for RSV than for the other vaccines, appropriately reflecting FDA/CDC approval for pregnancy administration only in the 32°0 - 36°6 week window ^40^. 1/31 mothers had possible influenza infection in pregnancy (treated presumptively with Tamiflu based on classical symptoms during influenza season); no mothers reported symptomatic RSV infection in pregnancy. No infants exhibited signs or symptoms of clinical RSV or other respiratory viral infection in the first two months of life; one infant had self-limited wheezing of unknown etiology. All mothers reported breastfeeding in the first week of life; 28/31 mothers reported breastfeeding at 2 months of life.

### RSV specific immunoglobulins and their subtypes are differentially transferred across the placenta

The RSV vaccine induced a robust anti-RSV PreF IgG response that was dominated by IgG1 over IgG2-4 (Figure 1A). Anti-RSV PreF IgG and IgG1 were significantly higher in cord and 2-month infant blood than maternal blood (Figure 1A). Anti-RSV PreF IgG2-4 were present in maternal and cord serum at relatively lower levels than IgG1 (Figure 1A). IgM levels were significantly lower in cord than maternal blood because this isotype does not cross the placenta^42^, but were noted to increase in two-month infant samples (Figure 1A), possibly reflecting transfer through breastmilk or asymptomatic infant exposure, given that no parents reported clinically evident respiratory infection of these infants in the first two months of life.

To quantify transfer efficiency, maternal-to-cord transfer ratios (TR) were calculated, with values greater than 1 indicating the expected efficient transfer of vaccine-induced antibodies ^43^. Total IgG was efficiently transferred with an average TR of 1.3 (Figure 1B). Consistent with previous reports of preferential subclass transfer (IgG1 > IgG3 > IgG4 > IgG2)^43^, IgG1 and IgG3 displayed the greatest transfer efficiency (TRs of 1.2 and 1.3, respectively) (Figure 1B). In contrast, IgG2 and IgG4 were less efficiently transferred, with TRs of 0.9 and 1.1, respectively (Figure 1B). Numbers of participants with TR > 1 for each IgG isotype, reflecting efficient transfer, are depicted in Figure 1B, reflecting proportionately high transplacental transfer of maternal IgG but not maternal IgM (Figure 1C). Cord blood was defined by high levels of IgG, with IgG1 and IgG3 being the dominant subclasses, highlighted in polar plot analysis (Figure 1D). IgG1, IgG2, and IgG4 were more persistent in 2-month infant blood than IgG3, despite the more efficient transfer of IgG3 (Figure 1E). These analyses demonstrate efficient transfer of RSV PreF-specific IgG, particularly IgG1 and to a lesser extent IgG3, with more rapid waning of IgG3 relative to other IgG isotypes in infants by 2 months of age.

As maternal RSV vaccines contain only the PreF protein, glycoprotein G-specific antibodies can be used as a proxy for natural infection^44^, particularly as infection fails to confer long-lasting immunity^45,46^. Anti-RSV A and B G IgG levels were low across all isotypes in all blood sample types (**Supplemental Figure 1A-B)**. RSV A and B G protein-specific IgM was greater in maternal than cord or infant blood samples **(Supplemental** Figure 1A–B), reflecting a lack of placental transfer of IgM. Overall, these findings highlight important reshaping of the maternal and neonatal vaccine-generated antibody repertoire by transplacental transfer.

### RSV PreF FcγR-binding antibodies are efficiently transferred across the placenta

Anti-RSV PreF IgG binding FcγRIIAR, FcγRIIB, FcγRIIIAF, and FcγRIIIB exhibited higher levels in cord blood than maternal blood, with mean transfer ratios > 1 (Figure 1F–G). Levels of FcRn-binding antibodies were relatively similar between maternal and cord blood (Figure 1G). Analysis of transfer ratios demonstrated enrichment of FcγR-binding IgG in cord blood with average TRs of: FcγRIIIAF (1.12), FcγRIIAR (1.09), FcγRIIB (1.12), FcγRIIIAV (1.32), and FcγRIIIB (1.12) (Figure 1G). There was greater heterogeneity in levels of FcγRIIIAV binding antibodies relative to other FcγR-binding antibodies. Both maternal and cord blood were dominated by FcγR binding antibodies (Figure 1G–H), with the lowest proportions of FcR binding antibodies in 2-month infant blood (Figure 1J). FcRn was most prominent in maternal blood, contributing less to both cord and infant blood binding profiles (Figure 1G–J). FcγRIIIAV remained prominent in the 2-month infant profile despite showing high variability at birth (Figure 1J).

### H1 antibody isotypes and Fc binding affinity profiles are differentially transferred across the placenta

Influenza vaccines target the hemagglutinin (HA) surface protein of 4 distinct influenza viruses in the 2023 and 2024 seasons. For the purposes of these analyses, we focused on H1-specific antibodies. Anti-H1 IgG responses were greater in cord blood than in either maternal or two-month infant blood (Figure 2A). Anti-H1 IgG responses were dominated by IgG1 (Figure 2A), with lower quantities of IgG2-4 (Figure 2A). H1-specific IgG1 declined by two months of age relative to cord blood (Figure 2A), whereas levels of anti-RSV PreF IgG1 were relatively preserved from birth (cord blood) to two-month infant blood (Figure 1A). Anti-H1 IgM was not transferred across the placenta in significant quantities but was increased in select two-month infant samples relative to cord blood, consistent with either asymptomatic respiratory viral exposures, transfer in breastmilk, or both (Figure 2A). Maternal-to-cord TR demonstrated efficient transfer of H1-specific IgG, with average TR of 1.6 for total IgG and 2.0 for IgG1 (Figure 2B). Maternal levels of anti-H1 IgG2, IgG3, and IgG4 were relatively lower than total IgG and IgG1; despite lower starting maternal levels, efficient transfer was preserved with average TR of 0.96 for IgG2, 1.04 for IgG3, and 1.1 for IgG4 (Figure 2B). Maternal blood was characterized by proportional dominance of poorly placentally transferred antibodies, including anti-H1 IgM, IgG2, and IgG3 (Figure 2C). Anti-H1 total IgG and IgG1-4 dominated cord blood (Figure 2D) and at two months of age, anti-H1 IgG1, IgG4, and IgM were the dominant components of the infant antibody repertoire, reflecting greater persistence of IgG1 and IgG4 relative to IgG2 and IgG3, and either colostrum-acquired or infant exposure-acquired IgM (Figure 2E).

Like anti-RSV PreF antibodies, anti-H1 antibodies with FcγR affinity were efficiently transferred across the placenta and were significantly higher in cord blood relative to both maternal and infant blood (Figure 2F). The placental TR of anti-H1 IgG with affinity for FcγRs were all >1, with average transfer ratios of 1.3 for both FcγRIIAR and FcγRIIIAF, 1.33 for FcγRIIB, 1.47 for FcγRIIIAV, and 1.2 for FcγRIIIB (Figure 2G). Proportionally, anti-H1 IgG binding to FcγRs in cord blood was greater than in maternal or infant blood (Figures 2H–J). As with anti-RSV antibody profiling, FcRn-binding IgG was less efficiently transferred than FcγR-binding IgG, as evidenced by higher proportion of FcRn-binding antibodies in maternal blood and lower proportion in cord blood relative to FcγR-binding antibodies (Figures 2H–J). Greater relative persistence of FcγR-binding compared to FcRn-binding was noted in 2-month infant blood, as well (Figure 2J). As antibodies with different antigen-specific qualities were transferred across the placenta with different efficiencies, we examined whether functional antibody properties were selectively transferred.

### Transferred RSV-specific antibodies retain neutralizing capacity and Fc-effector functions in cord blood

Neutralizing antibodies (nAb) to RSV Type A and Type B were effectively transferred across the placenta (average TR= 1.6 for Type A and 1.5 for Type B) (Figure 3A–C). Given the observed enrichment of FcγR-binding antibodies in cord blood (Figure 1F), we next performed comprehensive functional profiling to determine whether RSV-specific antibodies in maternal and cord blood could effectively engage Fc-mediated effector pathways and recruit innate immune cells for antiviral defense. Antibody-dependent complement deposition was highly enriched in cord blood relative to maternal blood (Figure 3D), with 11 dyads having TRs >8 (Figure 3A).

To enable simultaneous evaluation of multiple Fc-mediated antibody functions, we developed a multiplexed functional assay coupled with expanded flow cytometric phenotyping of FcγR^+^ immune cell subsets **(Supplemental Figure 2 – gating strategy, Supplemental Table 2 - antibodies).** Unlike RSV neutralization and ADCD, RSV PreF ADNP exhibited FcγR-specific differences following placental transfer. While total ADNP did not differ between maternal and cord blood (Figure 3E), CD16^+^ (i.e., FcγRIII) neutrophils exhibited significantly greater phagocytic activity in cord than maternal blood, whereas CD32^+^ (i.e., FcγRII) neutrophils did not (Figure 3F–G). These data suggest selective placental transfer of anti-RSV PreF IgG binding FcγRIII on neutrophils to drive the phagocytic capacity of these cells.

The ability of anti-RSV PreF IgG to induce ADCP was greater in cord than maternal blood (Figure 3H), but this was not specific to anti-RSV PreF IgG binding to either CD16 or CD32 on monocytes (Figure 3I–J), suggesting that these responses might be mediated by binding to other FcγRs or FcRn. Unlike complement deposition and phagocytic functions, antibody-dependent NK cell cytotoxicity (ADNK) was lower in cord than maternal blood (Figure 3A). Anti-RSV PreF antibodies induced significantly lower frequencies of CD107a^+^, IFNγ^+^, and polyfunctional CD107a^+^IFNγ^+^ NK cells in cord than maternal blood among both CD16^+^ and CD32^+^ NK cell populations (Figure 3K–P), with <10/31 dyads having placental transfer ratios >1 for any ADNK phenotype (Figure 3A). These findings suggest that placentally-transferred antibodies generated by the maternal RSV vaccine have reduced capacity to drive NK cell-mediated effector responses.

To validate the multiplexed functional assay platform, well-established singleplex **(Supplemental Figure 3 – gating strategy)** ADCP, ADNP, and ADNK assays ^35,47,48^ were performed independently on a subset of samples and yielded highly concordant results **(Supplemental Figure 4A-F)**. Significant positive correlations were observed between multiplexed and singleplex measurements for ADCP (R = 0.6730), ADNP (R = 0.7197), and ADNK (R = 0.6539)**(Supplemental Figure 4G-I)**.

### Placental H1 antibodies are capable of neutralization and select FcR-mediated non-neutralizing functions

Neutralizing antibodies are the correlate of protection for influenza vaccines^49,50^. H1N1 nAbs were efficiently transferred across the placenta, with an average transfer ratio of 2.1 (Figure 4A–B). The substantial inter-individual variability observed (SD = 1.90) likely reflected differences in the levels of preexisting immunity. Antibody-dependent complement deposition was higher in cord than maternal blood, with an average transfer ratio of 9.84 (Figure 4A–C). H1-specific antibodies transferred across the placenta had lower total (Figure 4D) and CD32^+^ (Figure 4E) ADNP activity than in maternal blood, with no differential ADNP activity observed among CD16^+^ neutrophils (Figure 4F).

H1-specific antibodies exhibited increased total ADCP in cord blood relative to maternal blood (Figure 4G) that was not mediated by either CD16^+^ or CD32^+^ monocytes. CD16^+^ monocytes exhibited reduced phagocytic activity in response to H1 antibodies from cord relative to maternal blood, whereas CD32^+^ monocytes exhibited no change in activity following exposure to either maternal or cord blood H1 antibodies (Figure 4H–I). H1-specific antibodies displayed a heterogeneous ADNK profile. Within CD16^+^ NK cells, only IFNγ production was reduced in cord compared with maternal blood, whereas CD107a expression and polyfunctional CD107a^+^IFNγ^+^ responses were equally maintained in response to maternal and cord blood antibodies (Figure 4J–L). Among CD32^+^ NK cells, antibody-mediated degranulation (CD107a^+^) was increased in cord relative to maternal blood, whereas IFNγ production and polyfunctional responses were reduced (Figure 4M–O). Placental transfer of H1 antibodies inducing diverse ADNK functions was less efficient than for any other antibody functions evaluated, with the exception of CD107a^+^ CD32^+^ADNK, which demonstrated efficient transfer (TR > 1) in 18/31 (Figure 4A).

### Steroids and cytokines are differentially transferred across the placenta

Soluble mediators, including cytokines and steroids, can regulate antibody secretion from B cells ^51–55^ which could impact antibody abundance and transfer across the placenta. Cytokines and chemokines were multiplexed and their relative concentration in cord blood versus maternal blood was quantified (Figure 5A–E). Cord blood contained significantly greater concentrations of IL-12 and IL-17A, whereas IL-10, IFN-α, and TNF-α were significantly lower in cord than maternal blood (Figure 5A–D). Cord versus maternal blood cytokine levels demonstrated substantial heterogeneity (Figure 5E), with the cytokines most consistently elevated in cord blood relative to maternal being IL-12 (average TR = 2.5), IL-17a (average TR = 9.5), IL-2R (average TR =1.13), and CCL4 (average TR = 1.6) (Figure 5E). Considerable inter-individual variability was noted for cord vs maternal levels, particularly for IL-17A and CCL4 (Figure 5E). Proportionally, maternal blood was enriched for IL-10 and IL-15, whereas the cord blood cytokine signature was dominated by IL-12 and IL-17A (Figure 5F–G).

To characterize the hormonal milieu, cortisol, estradiol, and progesterone concentrations were measured in matched maternal-cord blood pairs (Figure 5H–J). Cortisol concentrations were significantly greater in maternal than cord blood (Figure 5H). Estradiol concentrations were highly variable and did not differ between maternal and cord blood (Figure 5I). In contrast, progesterone concentrations were significantly elevated in cord relative to maternal blood (Figure 5J), consistent with previous studies highlighting the abundance of progesterone found in cord blood^56^.

### Distinct immune features are associated with persistence of RSV- and H1N1-specific antibodies during early infancy

Whether heterogeneity in the concentrations of cytokines or steroids could influence antibody transfer and durability was explored using machine learning models to predict the durability of antigen-specific IgG1in infant circulation at two months of age. We applied sparse partial least squares discriminant analysis (sPLS-DA) to integrate antibody, cytokine, and steroid hormone datasets generated from matched maternal and cord blood samples. Models were trained to distinguish infants with high versus low RSV PreF-specific (cutoff: 3.894 log_10_[MFI]) or H1-specific (cutoff: 3.022 log_10_[MFI]) IgG1 at two months of age (Figure 6A–B), as IgG1 was the predominant subclass induced by both vaccines and exhibited the greatest placental transfer efficiency. Both sPLS-DA models demonstrated moderate classification performance (RSV error rate = 44.0%; H1N1 error rate = 37.3%) and identified distinct plasma features associated with antibody persistence (Figure 6C–D). Despite differences between vaccine antigens, estradiol emerged as a selected feature in both models. For RSV, cord blood total IgG abundance, ADCP, and estradiol in maternal blood were contributors to high infant IgG1 persistence **(Supplemental Figure 5A-C)**. In contrast, maternal blood ADCP and cord blood RSV A2-specific neutralizing antibody responses were associated with low infant IgG1 durability **(Supplemental Figure 5D-E)**. Cord blood estradiol was the only feature associated with greater H1-specific IgG1 in infants **(Supplemental Figure 5B, F)**. For H1N1, maternal progesterone, maternal ADNP, and both maternal and cord H1-specific IgG2 were associated with lower infant IgG1, **(Supplemental Figure 5G-J)**. These data suggest a possible role for maternal and placental estradiol in predicting greater antiviral immunity in infants.

## Discussion

Maternal vaccination generates antibodies that undergo functionally selective placental transfer, resulting in enrichment of antibody quality rather than simply antibody abundance. In addition to preferential transfer of vaccine antigen-specific IgG1, cord blood anti-RSV PreF and H1 antibodies exhibited enhanced binding to FcγRs, including FcγRIIA, FcγRIIIAF, FcγRIIIAV, and FcγRIIIB, accompanied by enrichment of ADCD and ADCP effector functions. This selective enrichment is consistent with previous studies demonstrating preferential transfer of FcR-binding antibodies following influenza and tetanus, diphtheria, and pertussis (Tdap) vaccination, as well as reports of digalactosylated Fc glycans in cord blood after maternal Tdap immunization, a glycosylation profile associated with enhanced FcγR engagement and placental transfer^34,57,58^. Our findings support a hierarchical model of placental antibody selection in which antibody subclass, FcR engagement, and effector function are enriched, which could serve to maximize the protective potential of maternally derived antibodies during early life.

The preferential enrichment of FcR-mediated antibody functions in cord blood may reflect an adaptation to the unique immunological landscape of early life. During the neonatal period, protection against viral infection relies heavily on maternally derived antibodies because adaptive immune responses and immunological memory remain immature^59^. Transfer of antibodies that efficiently neutralize or induce innate immune effector mechanisms may provide a substantial advantage during the first months of life. Placental transfer preferentially preserved neutralizing, complement-activating, and phagocytic antibody functions, whereas CD107a^+^ and IFNy^+^ NK cell-activating antibodies were comparatively underrepresented in cord blood. Neonatal monocytes largely retain phagocytic capacity ^60,61^, supporting ADCP as an effective antiviral mechanism early in life, whereas neutrophils and NK cells exhibit impaired phagocytic and cytotoxic functions in neonates ^62–67^. Although concentrations of many complement proteins are lower in neonates than in adults, the neonatal complement system remains functionally active, suggesting that reduced complement availability does not preclude complement-mediated effector functions at birth^68,69^. These observations suggest that placental antibody transfer may not only select for neutralization, but also for FcR engagement and innate immune cell effector functions that are best suited to the neonatal immune environment.

Existing studies of maternal vaccination have demonstrated efficient placental transfer of antibodies with enhanced FcγR binding and multiple Fc-mediated functions, including ADCP, ADNP, and ADCD^70^. Across studies, neutralizing, phagocytic, and complement-mediated functions appear to consistently transfer with high efficiency, whereas NK cell-mediated functions show greater variability depending on the immune context and functional readout ^26,27,34,71,72^. In the current study, RSV specific antibodies transferred across the placenta exhibited reduced ADNK activity in cord compared with maternal blood, whereas influenza-specific antibodies showed greater variability in the transfer of ADNK activity across functional readouts. Existing data show influenza vaccination increased ADNK activity in maternal and cord blood in comparison to unvaccinated pregnancy women; TR, however, were lower in the vaccinated group, suggesting inefficient transfer of ADNK activating antibodies after vaccination during pregnancy^73^. In a study comparing COVID-19 vaccine platforms, both vaccine platform and trimester of maternal vaccination influence generation and transfer of non-neutralizing functional antibodies, with first trimester vaccination and mRNA vaccine platforms resulting in the most efficient antibody transfer^48^. Findings from the current study suggest that placental transfer may more consistently preserve neutralizing, phagocytic, and complement-mediated functions, while NK-cell-mediated functions are more context-dependent.

Although similar functional enrichment was observed following both maternal RSV and influenza vaccination, important antigen-specific differences emerged. While RSV PreF-specific antibodies in cord blood induced increased CD16-dependent neutrophil phagocytosis following placental transfer, H1-specific antibodies caused reduced ADNP overall and among CD32^+^, but not CD16^+^, cells while also maintaining CD107a^+^ and polyfunctional CD107a/IFNγ^+^ NK cell populations. These differences indicate that the functional programming of transferred antibodies is influenced by antigen-specific characteristics rather than reflecting a universal mechanism of placental selection.

One potential explanation for antigen-specific functional differences is the distinct immunological context in which these vaccines are administered. Because of repeat exposures and pre-existing immunity, seasonal influenza vaccination predominantly recalls established memory B cell responses^74–76^. RSV vaccination is similarly administered in the presence of pre-existing immunity, as RSV infection occurs repeatedly throughout life and nearly all pregnant individuals have detectable RSV-specific antibodies prior to vaccination^77,78^. Pre-existing RSV antibody levels, however, may be relatively low and may exert less influence on the vaccine response than the higher-titer, repeatedly boosted influenza-specific antibody repertoire. Differences in the magnitude, specificity, and maturation of pre-existing immunity, rather than its presence or absence, may contribute to the distinct functional antibody profiles elicited by influenza and RSV vaccination.

IgG1 comprises approximately 90% of total IgG antibodies. This isotype is the most functionally diverse IgG subclass, exhibits the longest half-life, robustly mediates both neutralizing and non-neutralizing antibody functions, and is a critical component of a competent antibody repertoire^79,80^. Supervised machine learning identified distinct immune and steroid features associated with sustained infant IgG1 responses for RSV and H1N1, suggesting that persistence is regulated through different mechanisms for different vaccine antigens. For anti-RSV PreF IgG1, persistence was associated with characteristics of the transferred antibody repertoire, including cord blood total IgG abundance and ADCP, linking Fc-mediated antibody quality with the durability of passive immunity. Antibodies capable of mediating ADCP generally exhibit favorable Fc receptor interactions, subclass composition, and Fc glycosylation profiles^30^. Together with the sustained function of neonatal monocytes, data from mouse models suggest that monocytes may be necessary components of antibody-mediated antiviral immunity^81^. In contrast, persistence of H1-specific IgG1 in infants was associated primarily with lower maternal progesterone at delivery and antibody subclass features (e.g., IgG2), consistent with distinct determinants of influenza-specific antibody maintenance^82^. H1-specific antibody persistence may be influenced more by endocrine and antibody-intrinsic characteristics than by Fc-mediated effector function. Estradiol emerged as the strongest positive contributor to the durability of infant IgG1 in both models. Estrogen has well-established effects on B cell responses^52,53,83^, Fc glycosylation^39,84^, and placental physiology^85^, suggesting that the endocrine environment during late pregnancy may contribute to both placental antibody transfer and the persistence of maternally derived antibodies in infancy. While this association may reflect overfitting and requires validation in larger cohorts, the consistent observation of estradiol as a stimulator of greater infant IgG1 across both vaccine antigens suggests that maternal hormones may influence passive immunity beyond effects on antibody concentration alone.

This study highlights that evaluating maternal vaccines using antibody concentrations or neutralizing activity alone is likely insufficient to define correlates of infant protection. Instead, FcR engagement, FcR-mediated effector functions, and antibody persistence represent complementary dimensions of passive immunity that likely contribute to neonatal protection. There should be greater consideration of the preferential transfer of antibodies that activate complement activity and monocyte phagocytosis. This concept also may extend to passive immunization strategies. RSV monoclonal antibodies, including nirsevimab and clesrovimab, are engineered as IgG1 antibodies to maximize persistence; their protective activity, however, has been characterized primarily through neutralization, and comparatively little is known about their capacity to engage diverse FcR-mediated effector functions^15^. Limited available data suggest that polyclonal antibodies induced by maternal vaccination are expected to have a more favorable glycan and FcR-binding profile promoting diverse antibody effector functions, relative to the engineered monoclonal antibodies nirsevimab and clesrovimab^29,86,87^. Both nirsevimab and clesrovimab carry the YTE mutation, which is known to reduce FcγRIIA, FcγRIIB, and FcγRIIIA receptor engagement in favor of FcRn binding^88^. Recombinant monoclonal antibodies also are typically core-fucosylated, which can be associated with reduced FcγRIIIA affinity, whereas vaccine-induced polyclonal responses may enrich for more heterogeneously glycosylated antibodies, including but not limited to afucosylated antibodies with higher FcγRIIIA engagement potential^29,86^. More comprehensive evaluations of antibody quality and glycosylation profiles may prove critical to improving both maternal vaccine development and the design of next-generation antibody-based interventions for infant protection.

This study has limitations. The study cohort was modest in size and was not designed to directly compare vaccine immunogenicity between RSV and influenza vaccination, nor was it powered to disaggregate and consider infant sex in analyses of results. Although the comparative analyses revealed distinct antigen-specific patterns of antibody transfer and persistence, these observations require validation in larger, independent cohorts where influenza vaccines are administered during the same gestational window as RSV vaccines and with ample numbers to appropriately consider infant sex. Infant antibody persistence was evaluated at a single postnatal time point. Future studies incorporating longitudinal infant follow-up will be important to define the durability and clinical significance of the functional antibody profiles. We did not characterize antibodies transferred through breastmilk, which likely influences the infant antibody repertoire and protection after birth. Future studies integrating placental transfer, breastmilk immunity, antibody glycosylation, and clinical outcomes will provide a more comprehensive understanding of passive immunity following maternal vaccination.

In summary, this study demonstrates that placental transfer selectively enriches the functional quality of vaccine-induced antibodies, shaping the repertoire of passive immunity delivered to and maintained in the infant. Evaluating features of antibodies generated by maternal vaccines beyond titer/concentration and neutralization can reveal key features of antibody function and persistence that are likely to influence infant protection. Incorporating multidimensional systems serology and hormonal measurements into maternal vaccine evaluation may improve the identification of correlates of protection and guide the development of next-generation maternal vaccines that optimize the functional quality and durability of antibodies transferred during pregnancy.

## Methods

### Study Design and Participants

This study reflects a cohort of pregnant women (n = 31) immunized with the maternal RSV vaccine Abrysvo during the third trimester, enrolled at Massachusetts General Hospital between October 4, 2023 and February 29, 2024 (Mass General Brigham IRB #2023P001874). Their infants were enrolled between January 19, 2024 and May 3, 2024 (MGB IRB #2020P000955). All participants received the seasonal quadrivalent (QIV) influenza vaccine as part of routine prenatal care, with the H1N1 pandemic 2009 like virus component being A/Wisconsin/67/2022. Eligible participants were: (1) pregnant or ≤2 months postpartum, (2) ≥18 years of age, (3) able to provide informed consent, and (4) recipients of seasonal QIV and Abrysvo during pregnancy. Matched maternal and umbilical cord blood samples were collected at delivery (n = 31). Infant blood was collected at 2 months of age using the Touch Activated Phlebotomy (TAP II) microneedle capillary blood collection device (YourBioHealth, Medford, MA). Blood was centrifuged at 1000 × g for 10 minutes at room temperature; plasma was aliquoted into cryogenic vials and stored at −80°C until analysis. Participant demographics and vaccination schedules are described in Table 1. All participants provided written informed consent prior to enrollment.

## Supplementary Material

This is a list of supplementary files associated with this preprint. Click to download.

• JHU117SupplementalInformation.docx

## Figures and Tables

**Figure 1:**
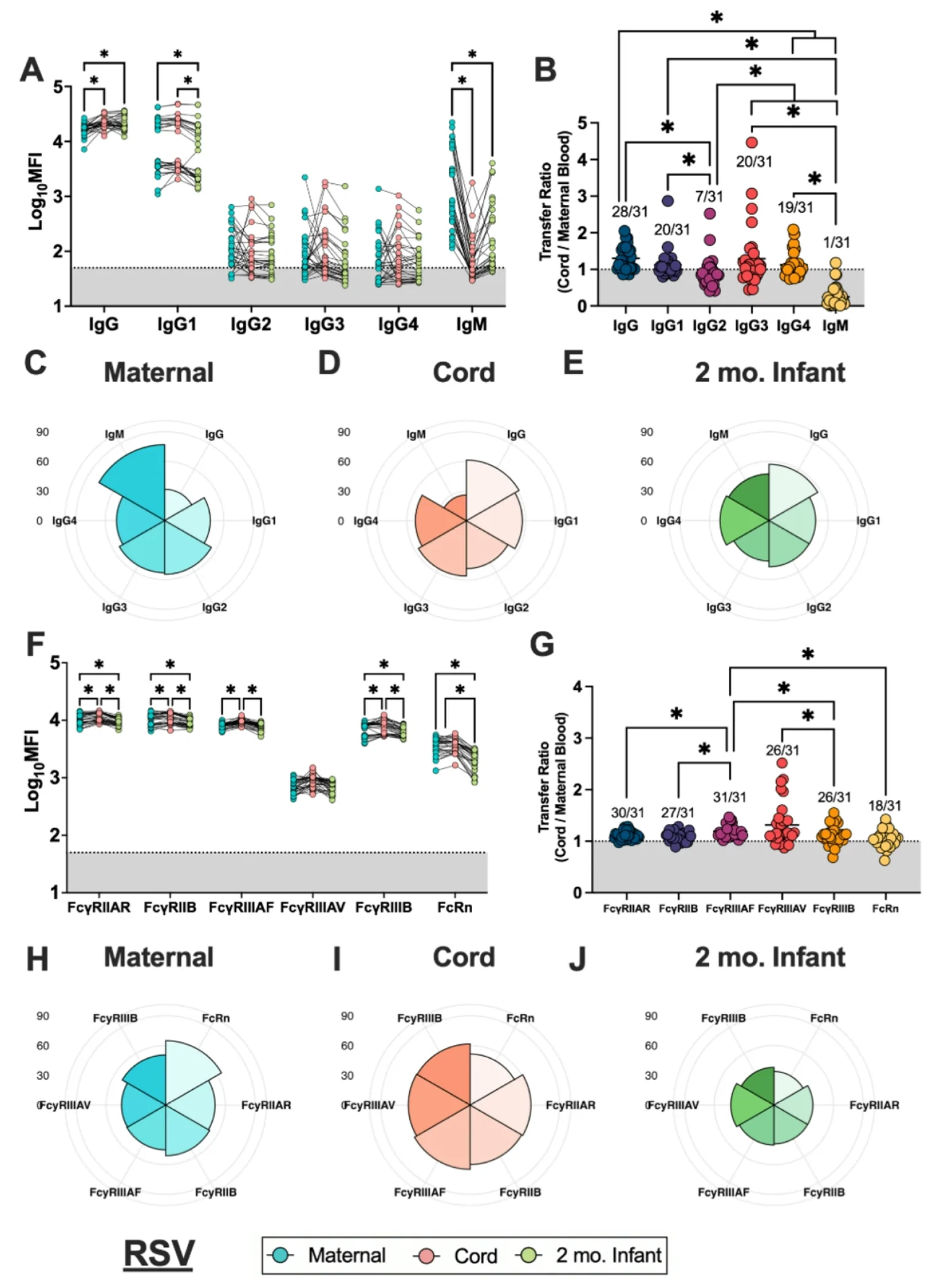
RSV PreF-specific immunoglobulin responses and Fc receptor (FcR) binding affinity was measured in serum from maternal blood (MB), cord blood (CB) and 2-month infant blood by systems serology and reported as log_10_ mean fluorescence intensity (MFI). Each line connects a matched samples from the same dyad. (**A**) Relative abundance of immunoglobulin isotypes (IgG, IgG1-4, IgM) were measured for all samples. (**B**) Ratio of CB/MB was reported as a transfer ratio for IgG and its subtypes with ratios indicating number of participants with transfer ratios > 1. (**C–E**) Polar plots show the average percentile rank (i.e., relative abundance) of RSV PreF-specific antibody isotypes and subclasses in **(C)** maternal blood, **(D)** cord blood, and **(E)** infant blood at 2 months of age. (**F)** FcR binding affinity to FcγIIAR, FcγIIB, FcγIIIAF, FcγIIIAV, FcγIIIB and FcRn was measured in all samples. (**G)** Ratio of CB/MB was reported as a transfer ratio for FcγRs with ratios indicating number of participants with transfer ratios > 1. **(H–J)** Polar plots show the relative abundance of RSV PreF-specific antibodies with FcR affinity in **(H)** maternal blood, **(I)** cord blood, and **(J)** infant blood at 2 months of age. Data were analyzed by **(A-B, F-G)** linear mixed effects regression analysis with Bonferroni post-hoc. Asterisks indicate *p* < 0.05. **(A, F)** The stippled line and gray shading indicate the limit of background (LOB), defined as the log_10_MFI of a defined negative control. **(B, F)** Dotted horizontal line at ratio = 1.0 indicates equal maternal and cord concentrations. **(C-E, H-J)** Polar plot axes represent the percentage contribution of each antibody isotype/subclass to the total RSV PreF-specific antibody response.

**Figure 2:**
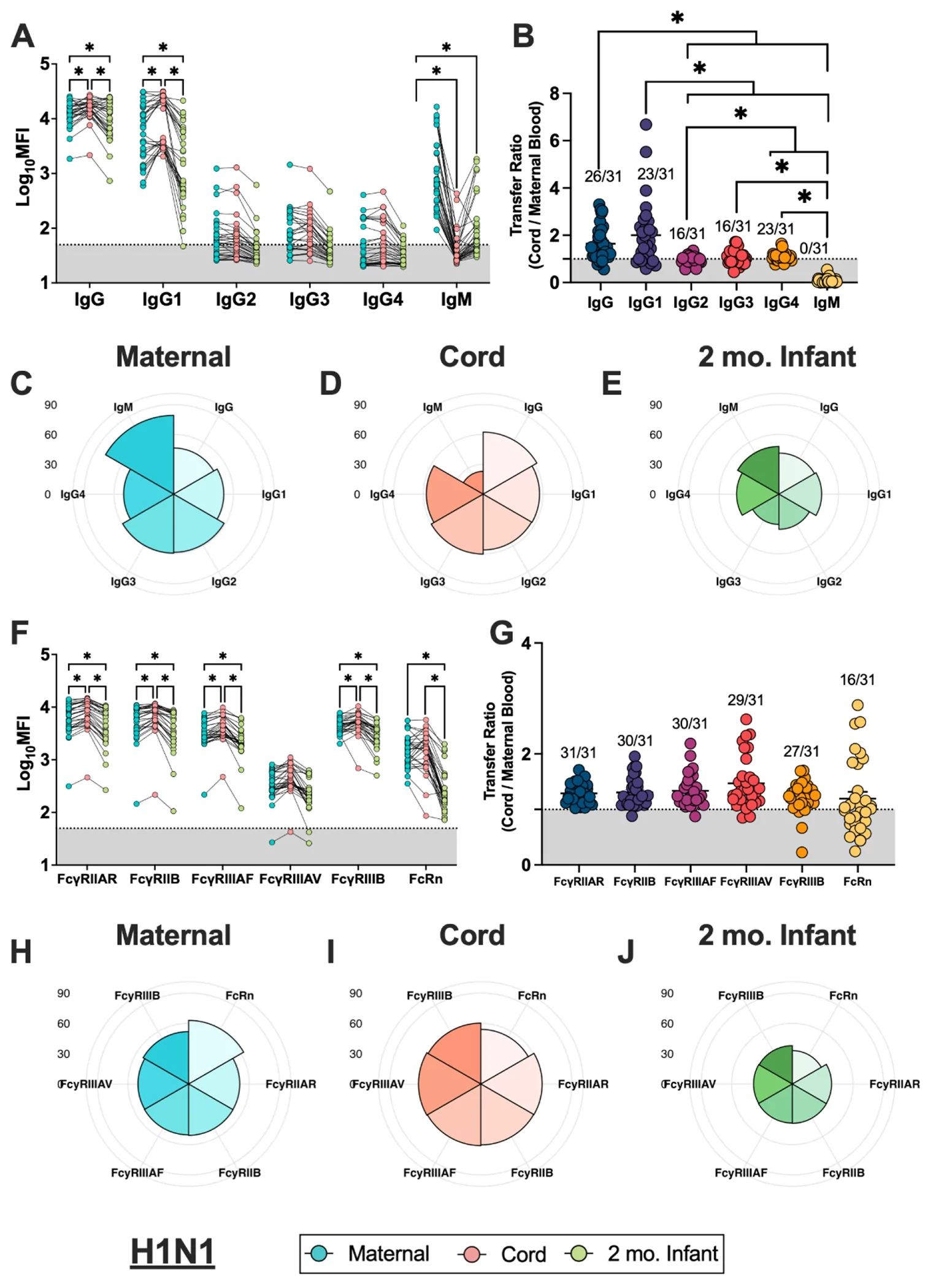
H1-specific immunoglobulin responses and Fc receptor (FcR) binding affinity was measured in serum from maternal blood (MB), cord blood (CB) and 2-month infant blood by systems serology and reported as log_10_ mean fluorescence intensity (MFI). (**A)** Relative abundance of immunoglobulin isotypes (IgG, IgG1-4, IgM) were measured for all samples. Each line connects a matched samples from the same dyad. (**B)** Ratio of CB/MB was reported as a transfer ratio for IgG and its subtypes with ratios indicating number of participants with transfer ratios > 1. **(C–E)** Polar plots show the average percentile rank (i.e., relative abundance) of H1-specific antibody isotypes and subclasses in **(C)** maternal blood, **(D)** cord blood, and **(E)** infant blood at 2 months of age. (**F)** FcR binding affinity to FcγIIAR, FcγIIB, FcγIIIAF, FcγIIIAV, FcγIIIB and FcRn was measured in all samples. (**G)** Ratio of CB/MB was reported as a transfer ratio for FcγRs with ratios indicating number of participants with transfer ratios > 1. **(H–J)** Polar plots show the average percentile rank (i.e., relative abundance) of H1 antibodies with FcR affinity in **(H)** maternal blood, **(I)** cord blood, and **(J)** infant blood at 2 months of age. Data were analyzed by **(A-B, F-G)** linear mixed effects regression analysis with Bonferroni post-hoc. Asterisks indicate *p* < 0.05. **(A, F)** The stippled line and gray shading indicate the limit of background (LOB), defined as the log_10_MFI of a defined negative control. **(B, G)** Dotted horizontal line at ratio = 1.0 indicates equal maternal and cord concentrations. **(C-E, H-J)** Polar plot axes represent the percentage contribution of each antibody isotype/subclass to the total H1-specific antibody response.

**Figure 3:**
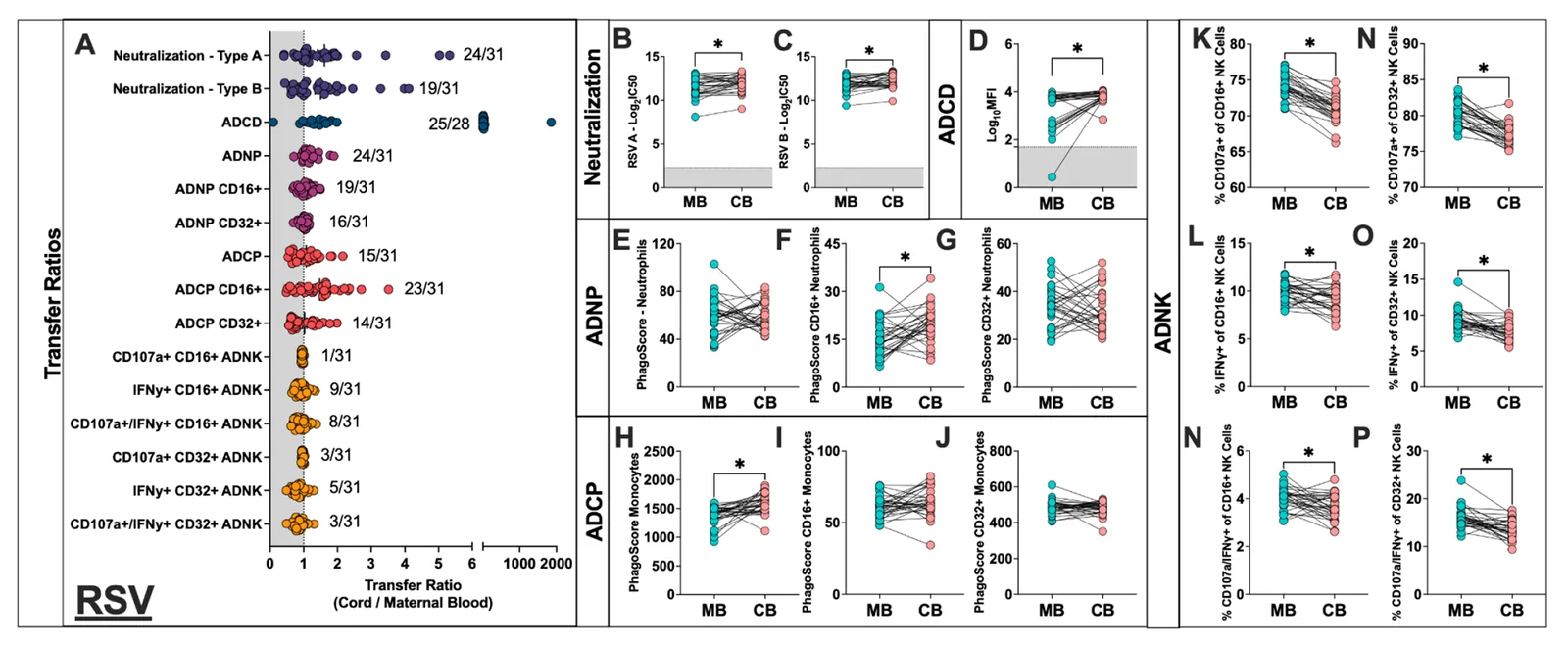
RSV PreF-specific antibody functions were measured in serum from maternal blood (MB) and cord blood (CB). Each line connects a matched maternal-cord sample from the same dyad. **(A)** Ratio of CB/MB was reported as a transfer ratio for neutralizing and non-neutralizing antibody functions with ratios indicating number of participants with transfer ratios > 1. Neutralizing antibody (nAb) was measured and reported as Log_2_IC50 for **(B)** RSV Type A and **(C)** RSV Type B. (**D)** Antibody dependent complement deposition (ADCD) was measured and reported as log_10_ mean fluorescence intensity. Cellular non-neutralizing functions were measured by multiplex flow cytometry analysis. Antibody dependent neutrophil phagocytosis (ADNP) and antibody dependent cellular phagocytosis (ADCP) were reported as PhagoScore, calculated as (% bead-positive monocytes/neutrophils) × (geometric MFI of each cell). Total phagoscore **(E, F)** was measured and further separated by FcgR positivity, including CD16^+^ **(F, I)** and CD32^+^ **(G, J)**. Antibody dependent NK cell (ADNK) cytotoxicity was measured by CD107a^+^, IFNγ^+^ and polyfunctional CD107a/IFNγ^+^, again separated by CD16^+^ **(K-M)** and CD32^+^ **(N-P)** NK cells. Data were analyzed by paired t-test. Asterisks indicate *p* < 0.05. The stippled line indicates the limit of background (LOB), defined as the log_10_MFI of a defined negative control.

**Figure 4:**
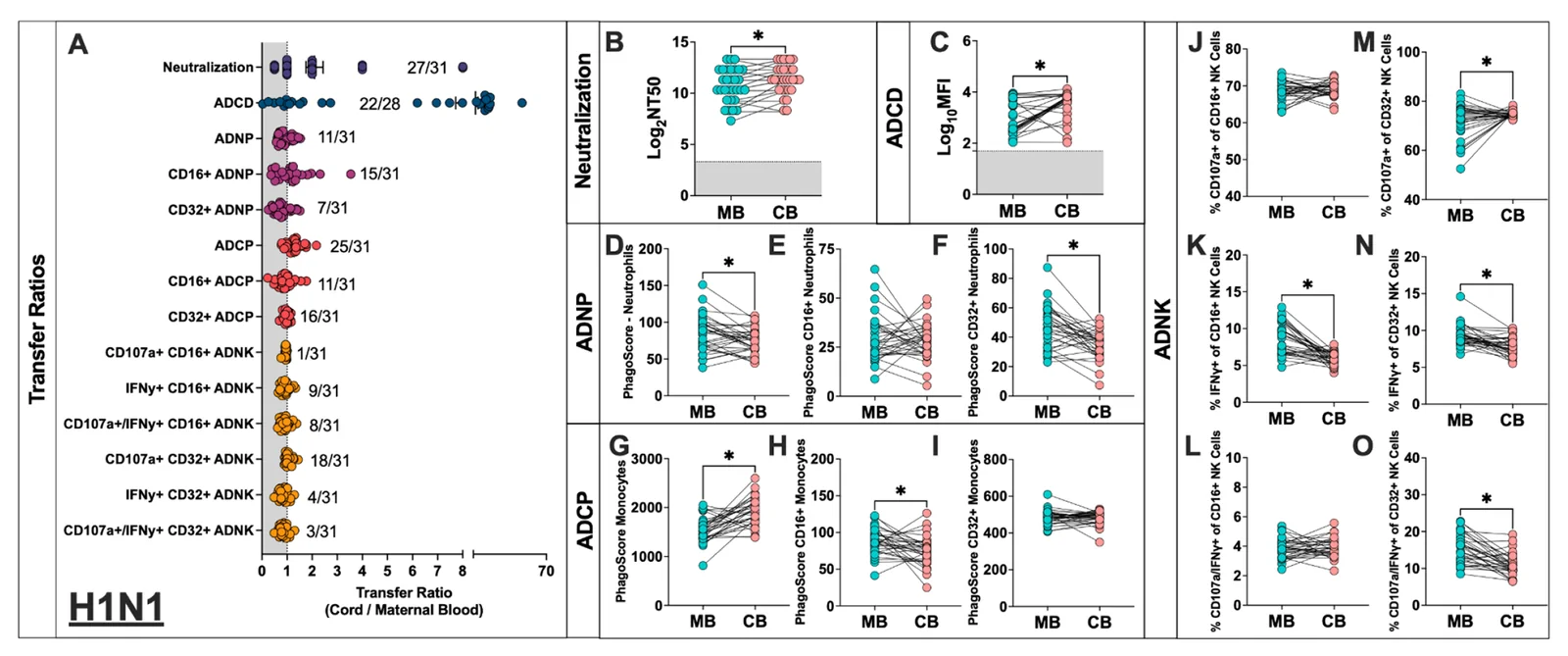
H1-specific antibody functions were measured in serum from maternal blood (MB) and cord blood (CB). Each line connects a matched maternal-cord sample from the same dyad. **(A)** Ratio of CB/MB was reported as a transfer ratio for neutralizing and non-neutralizing antibody functions with ratios indicating number of participants with transfer ratios > 1. **(B)** Neutralizing antibody was measured and reported as Log_2_IC50. (**C)** Antibody dependent complement deposition (ADCD) was measured and reported as log_10_ mean fluorescence intensity. Cellular non-neutralizing functions were measured by multiplex flow cytometry analysis. Antibody dependent neutrophil phagocytosis (ADNP) and antibody dependent cellular phagocytosis (ADCP) were reported as PhagoScore, calculated as (% bead-positive monocytes/neutrophils) × (geometric MFI of each cell). Total phagoscore **(D, G)** was measured and separated by FcR positivity including CD16^+^ **(E, H)** and CD32^+^ **(F, I)**. Antibody dependent NK cell (ADNK) cytotoxicity was measured by CD107a^+^, IFNγ^+^ and polyfunctional CD107a/IFNγ^+^, again separated by CD16^+^ **(J-L)** and CD32^+^ **(M-O)** NK cells. Data were analyzed by paired t-test. Asterisks indicate *p* < 0.05. The stippled line indicates the limit of background (LOB), defined as the log_10_MFI of a defined negative control.

**Figure 5:**
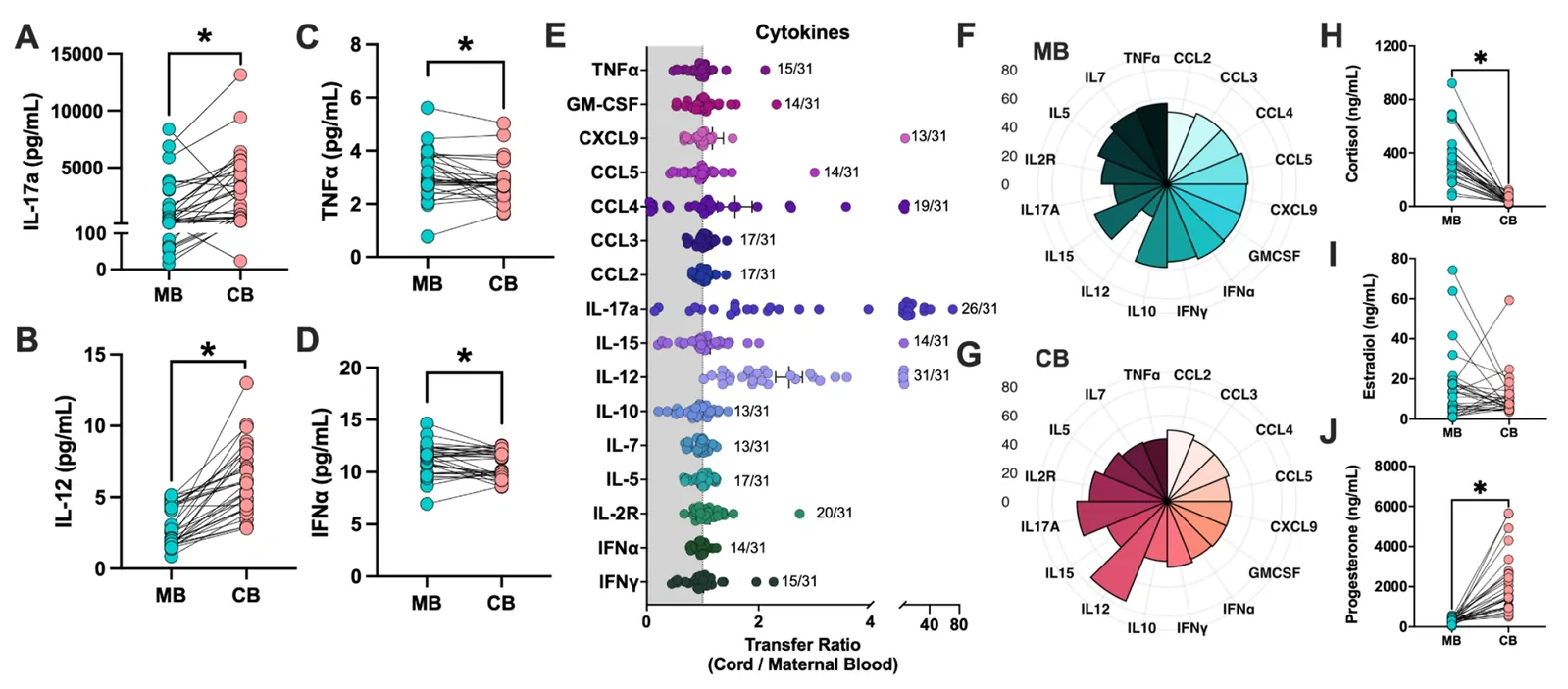
Matched maternal blood (MB) and cord blood (CB) samples from mother-infant dyads (n = 31) were analyzed for cytokine and chemokine concentrations by multiplex immunoassay. **(A-D)** Paired comparison of individual cytokines showing **(A)** IL-17, **(B)** TNFα, **(C)** IL-12, and **(D)** IFNα concentrations (pg/mL) in MB and CB. **(E)** Transfer ratios (CB/MB concentration) for 16 detectable cytokines and chemokines across all mother-cord dyads. Each point represents an individual dyad; horizontal bars indicate mean transfer ratios. Dotted horizontal line at ratio = 1.0 indicates equal maternal and cord concentrations. **(F-G)** Polar plots displaying average percentile rank of cytokine composition in **(F)** MB **(G)** and CB, illustrating the distinct cytokine profiles and immune microenvironments. Each spoke represents a different cytokine; spoke length indicates relative proportion of total cytokine signal. Hormone levels were quantified by Luminex-based immunoassay for the following hormones: **(H)** cortisol **(I)** estradiol and **(J)** progesterone. Each line connects a matched maternal-cord sample from the same dyad. Statistical significance was determined by paired t-test. Asterisks indicate *p* < 0.05.

**Figure 6:**
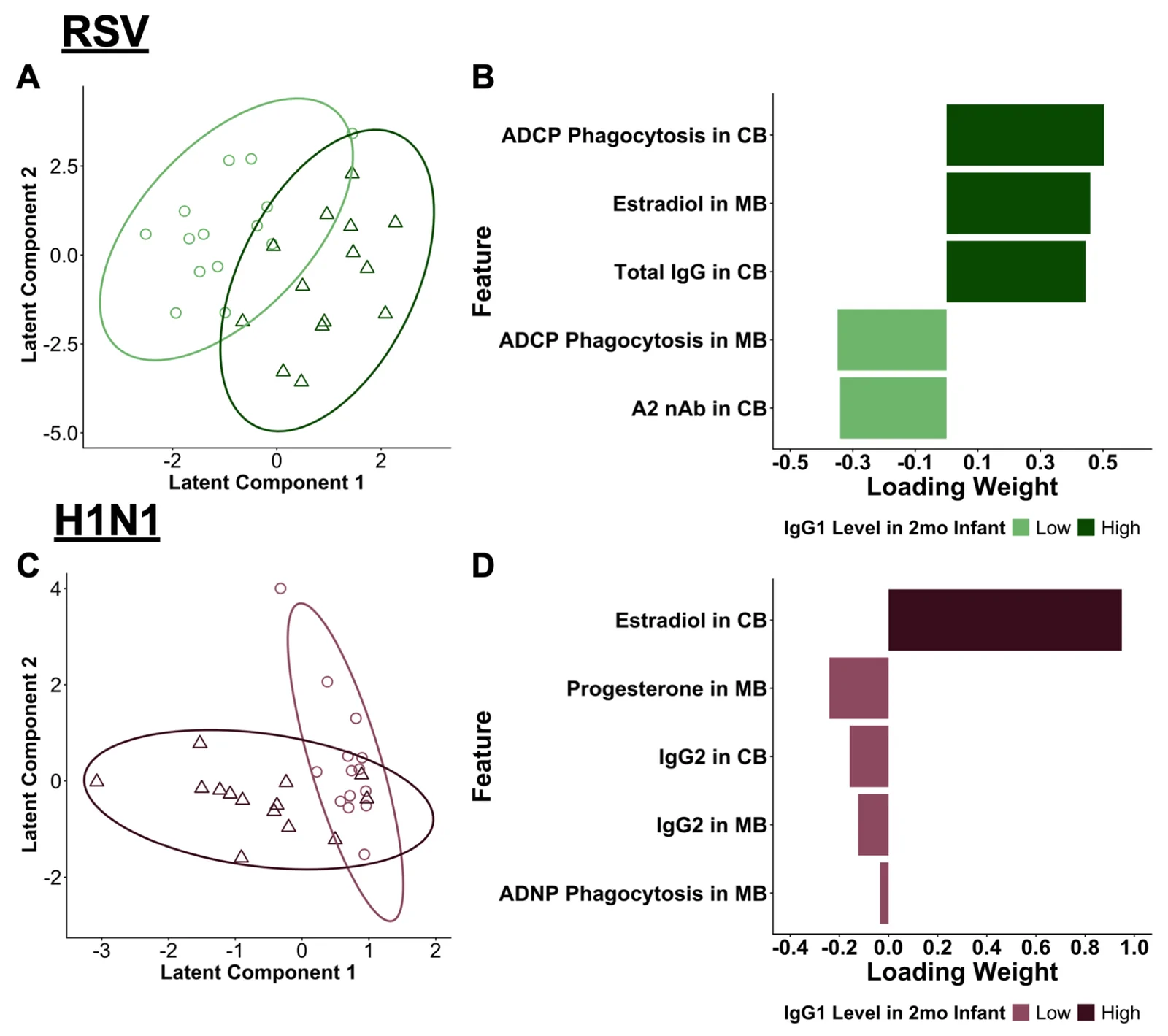
Sparse partial least squares discriminant analysis (sPLS-DA) integrating maternal blood (MB) and cord blood (CB) immune and steroid features stratified by high versus low 2-month infant RSV PreF-specific (green) or H1-specific (plum) IgG1 levels. **(A-B)** 95% confidence ellipses show group clustering distributions of the first two latent components, demonstrating partial separation between infants with high and low antibody persistence. **(C-D)** Feature loading weights contributing to the latent components to classify infant IgG1 levels (high or low), with features having higher loading weights being more important for classifying infant IgG1 levels. The coloring of bars represents the infant IgG1 group (high or low) that had the higher median value for each model-selected feature.

**Table 1.** Clinical Characteristics and Patient Demographics. A cohort of pregnant women (n = 3) receiving the Abrysvo RSV vaccine during the third trimester and the seasonal quadrivalent (QIV) influenza vaccine during any trimester, and their infants, were enrolled from October 2023 to May 2024.

Clinical Characteristics	N = 31

*Pregnancy Demographics*	Mean (SD)

Maternal Age (years)	35.6 (4.37)
Gestational Age of Delivery (weeks)	39.2 (1.12)
Birthweight (kg)	3.56 (0.48)

*Pregnancy Outcomes*	N (%)

Female Infant Sex	14 (45.2)
Infection During Pregnancy	
COVID-19	8 (25.8)
MRSA	1 (3.23)
Influenza	1 (3.23)

*Race*	N (%)

White	26 (83.8)
Black	1 (3.23)
Asian	3 (9.69)
Unknown	1 (3.23)

*Ethnicity*	N (%)

Hispanic or Latino	2 (6.46)
Not Hispanic or Latino	28 (90.3)
Unknown	1 (3.23)

*Vaccination During Pregnancy*	N (%)

RSV	31 (100)
Influenza	31 (100)
Tdap	31 (100)
COVID-19 (11 not vaccinated)	20 (65.5)

*GA at Vaccination (weeks)*	Mean (SD)

RSV	33.5 (1.33)
Influenza	22.6 (5.08)
Tdap	28.2 (0.93)
COVID-19 (11 not vaccinated)	25.6 (7.96)

*Days Between Vaccination & Delivery*	Mean (SD)

RSV	39.6 (9.69)
Influenza	115.7 (34.77)
Tdap	76.55 (10.30)
COVID-19 (11 not vaccinated)	95 (52.09)

## Data Availability

All anonymized data that support the findings of this study can be made available upon reasonable request.

## Code Availability

All code used for ML can be found at https://github.com/ayin0510/JHU117-Maternal-Vaccine-Induced-Transfer-of-Antigen-Specific-Antibodies.git.
